# Metagenomic Insights into the Enhancement of Bioavailable Nitrogen in Continuous Cropping Soil Through the Application of Traditional Chinese Medicine Residue Following Fumigation

**DOI:** 10.3390/genes15121532

**Published:** 2024-11-28

**Authors:** Xiangqin Xu, Xi Gao, Chen Gui, Hang Wang, Xiaowen Liu, Guoxing Wu

**Affiliations:** 1 State Key Laboratory for Conservation and Utilization of Bio-Resources in Yunnan, Yunnan Agricultural University, Kunming 650201, China; xiangqin_xu523@outlook.com (X.X.); chonchon@163.com (X.G.); 2 Chemistry and Bioengineering, Hunan University of Science and Engineering, Yongzhou 425199, China; gc18574643375@163.com; 3 National Plateau Wetlands Research Center, Southwest Forestry University, Kunming 650224, China; hwang17@163.com

**Keywords:** metagenome, TCMR, soil fumigation, the soil bioavailable nitrogen

## Abstract

**Background/Objectives:** Chemical fumigation can effectively inhibit the occurrence of soil-borne diseases; however, this approach can negatively affect the structure of the soil microbial community. The combination of soil fumigant and organic fertilizer application thus represents a widely adopted strategy in agricultural practice. Traditional Chinese medicine residue (TCMR) is a high-quality organic fertilizer; however, the impact of post-fumigation TCMR application on keystone taxa and their functional traits remains uncertain. **Methods:** This study examined the effects of five fertilization treatments on the diversity, key species, and related functional genes of microbial communities in rhizosphere soil of continuous cropping pepper. **Results:** Chemical fumigation followed by TCMR application markedly enhanced soil nutrient content in the rhizosphere and significantly influenced microbial community composition as well as functional gene patterns associated with microbial nitrogen cycling. It was also strongly correlated with soil bioavailable nitrogen content. The abundance of keystone bacterial species (Pseudomonadota, Actinomycetota, and Bacillota) substantially increased following TCMR application, alongside a notable rise in Ascomycota abundance within the fungal community. This shift contributed to an increase in beneficial bacterial abundance while reducing that of harmful bacteria. Additionally, TCMR addition affected the abundance of denitrification and DNRA genes involved in nitrogen cycling; specifically, *nirB* and *nirK* were strongly associated with soil organic nitrogen content. **Conclusions:** The combined application of chemical fumigants and TCMR modified the composition of keystone microbial community species by influencing rhizosphere soil TN and other nutrients, and these alterations were linked to multiple nitrogen-cycling functional genes.

## 1. Introduction

Microbial ecological imbalance in the rhizosphere is a major contributor to obstacles in continuous cropping systems, disrupting the interdependent ecosystem formed by plants, soil, and microorganisms. This imbalance exacerbates the degradation of the soil microenvironment, impairs plant health, and promotes the excessive proliferation of pathogens, ultimately leading to outbreaks of soil-borne diseases [1,2,3]. While soil fumigation is effective in mitigating soil-borne diseases, it adversely affects the structure of soil microbial communities, with different fumigants having varying impacts. For example, cottonseed fumigation reduces the populations of soil fungi, bacteria, and actinomycetes [4], whereas chloropicrin fumigation decreases the diversity of bacterial and fungal communities [5]. Research has demonstrated that combining soil fumigation with bio-organic fertilizers is an effective integrated pest management strategy. This approach suppresses pathogenic microbes while enhancing beneficial microorganisms and restoring the balance of microbial community to control diseases. Applying chemical fertilizers following fumigation can aid in the recovery of beneficial soil microorganisms and increase the mortality of pathogenic microbes [6]. Additionally, fumigation can directly inhibit fungal pathogens and, through the use of organic amendments, indirectly suppress both fungal and bacterial pathogens by altering microbial communities [6]. Therefore, the complementary use of organic amendments and soil fumigants offers a promising strategy for controlling soil-borne diseases. Assessing the impact of combined fumigation and organic fertilizer application on soil structure is essential for identifying green organic fertilizers that facilitate the recovery of soil microbial communities.

Traditional Chinese medicine residue (TCMR) refers to the solid plant-based residue remaining after the extraction of pharmaceutical constituents from medicinal materials, which contains valuable soil nutrients [7,8]. Conventional disposal methods such as incineration, landfill, and stacking face issues such as resource wastage and environmental pollution. Converting TCMR into high-value products represents an effective approach for resource utilization. China produces around 70 million tons of TCMR annually, which is sufficient to meet the demands of agricultural practices [9]. However, research on the effective use of TCMR in agricultural soils is still limited. TCMR contains numerous nutrients and active compounds, and it has the potential to improve the stability of soil microbial communities, enhance soil fertility, promote plant growth, and hold significant prospects for resource utilization and agricultural production [10,11]. Currently, in-depth investigations into the application of TCMR in agricultural continuous cropping soils remain scarce, and it is poorly understood whether TCMR can be effectively utilized as an organic fertilizer to enhance soil fertility post-fumigation, promote crop growth, and restore the microbial community in continuous cropping soils. Therefore, we hypothesized that (1) TCMR can alter soil nutrients, particularly nitrogen and organic matter, in continuous cropping soils; (2) the application of TCMR to fumigated soils can result in enhanced soil quality, suggesting its potential use as an organic fertilizer; and (3) providing a novel fertilization strategy, the application of TCMR in fumigated soil can improve the microbial community structure and increase species diversity related to nitrogen cycling genes. In this study, we employed the rhizosphere soil from pepper crops cultivated continuously for two years as the research subject to investigate the effects of TCMR application post-fumigation on crop rhizosphere soil nutrients, to explore the influences of TCMR application on the rhizosphere microbial community, and to examine the correlation between the microbial community and the soil physicochemcial properties.

## 2. Materials and Methods

### 2.1. Soil Sampling and Treatments

The study was conducted on rhizosphere soil (0–20 cm) from farmland near Hunan University of Science and Engineering (26°20′ N, 111°61′ E), where pepper had been continuously planted for 2 years. The soil was classified as yellow-cinnamon soil. The area has an average elevation of 1250 m, belongs to the transition zone between the temperate and tropical zones., with an average annual temperature of 18.0 °C, a frost-free period of 285–311 days, and an average annual rainfall of 1595 mm. In the second year of planting (7 November 2022), after the pepper harvest, rhizosphere soil was collected from a depth of 0–20 cm using a sampler. Five soil cores from the rhizosphere of the pepper plants were randomly collected from each region and thoroughly mixed to form a composite rhizosphere soil sample. Subsequently, any sand and plant residues were removed, and the samples were stored in airtight bags before being transported back to the laboratory in an incubator. The samples were sieved through a 2-mm sieve for rhizosphere soil microcosm experiments and analysis of rhizosphere soil physicochemical properties.

### 2.2. Soil Microcosm Experiments

Five treatment groups were established, including dazomet fumigation without TCMR application (M1, 300 kg/hm^2^, Jiangsu Qili New Energy Technology Co., Ltd., Taizhou, China), dazomet fumigation (300 kg/hm^2^) combined with TCMR (20 t/hm^2^) application (MC), 42% metam-sodium fumigation without TCMR application (W1, 400 kg/hm^2^, Shandong Lifan Chemical Industry Co., Ltd., Tai’an, China), 42% metam-sodium fumigation (400 kg/hm^2^) combined with TCMR (20 t/hm^2^) application (WC), unfumigated soil with TCMR application (C1, 20 t/hm^2^), with each treatment repeated three times. The TCMR from *Andrographis paniculata* is provided by Jinan Baishun Technology Co., Ltd. (Jinan, China), and SOM 46.68%, TN 1.45%, and P_2_O_5_ 1.18%. The TCMR was moistened and fermented with water for a week before application.

The absolute soil water content was adjusted to 21% before the incubation, corresponding to a water-filled pore space (WFPS) of 45%. Soil samples of 300 g each (dry weight) were packed into 500 mL Duran wide-mouth glass bottles (Schott AG, Mainz, Germany) to simulating natural ecosystems under human-controlled settings [12]. Chemical fumigants were added to the soil samples, while the control group received an equal volume of sterilized distilled water. The bottles were then sealed with stoppers and incubated at 28 °C. After 10 days of fumigation, the caps were removed, and the bottles were placed in a fume hood until the fumigants had completely dissipated. TCMR was subsequently added to the soil samples. Fresh air was introduced into all bottles using a pump, and the bottles were resealed and returned to the 28 °C incubator for an additional 59 days [13]. Remove the cap and mix the soil sample with a sampling spoon. The physicochemical parameters and molecular ecological measurements of the soil samples were analyzed. If analysis could not be conducted in a timely manner, the samples were stored in a refrigerator at −80 °C.

### 2.3. Soil Nutrient Determination

Soil water content (SWC) was determined by drying the soil at 105 °C for 12 h. The pH (soil/water = 1:2.5) was measured using a pH meter, and soil organic matter (SOM) was determined using the H_2_SO_4_-K_2_Cr_2_O_7_ oxidation capacity method. Soil total phosphorus (TP) and available phosphorus (AP) were determined using the molybdenum antimony colorimetric method. Total nitrogen (TN) was measured using the Kjeldahl method [14]. Soil nitrate (NO_3_^−^-N) and ammonium (NH_4_^+^-N) were extracted with potassium chloride and subjected to continuous flow analyzer analysis. Urease, catalase, sucrase, and neutral phosphatase activities were determined using a kit provided by Nanjing Jitest Biotechnology Co., Ltd. (Nanjing, China)

### 2.4. DNA Extraction and High-Throughput Sequencing

The Fsat DNA Spin kit (MP Biomedicals, Santa Ana, CA, USA) was used to extract microbial DNA from 0.5 g of fresh soil, and the concentration and purity of the extracted DNA were determined using a NanoDrop spectrophotometer (Thermo Scientific, Wilmington, NC, USA). Genomic DNA sample was fragmented by sonication to a size of 350 bp. Then DNA fragments were end-polished, A-tailed, and ligated with a full-length adapter for Illumina sequencing. Sequencing was performed on a NovaSeq 6000 platform (Illumina Inc., San Diego, CA, USA) at Wekemo Tech Co., Ltd., Shenzhen, China. Raw sequence data with an average of 3.31 Gb (gigabases) were obtained for each sample and deposited into the Genome Sequence Archive (GSA) with accession number PRJCA032586. Clean data were aligned to the host database using Bowtie2 (version 2.3.5.1, http://bowtie-bio.sourceforge.net/bowtie2/index.shtml, 14 July 2023) by default to filter out host-origin reads for subsequent analysis. The quality and effectiveness of the quality control process were assessed using FastQC (version 0.11.9, https://en.wikipedia.org/wiki/Fastq, 14 July 2023). Kraken2 (ver. 2.0.7-β) and the self-build microbial database (Sequences belonging to bacteria, fungi, archaea, and viruses were screened from NT nucleic acid database and RefSeq whole genome database of NCBI) were used to identify the species contained in the samples, and then Bracken was used to predict the actual relative abundance of species in the samples. The clean reads, after quality control and host removal, were used for blast against the database (Uniref90, https://www.uniprot.org/uniref?query=*, 25 July 2024) using Humann3 software (ver. 3.6) based on Diamond (ver. 0.8.22, https://github.com/bbuchfink/diamond, 25 July 2024). Statistical analysis of the relative abundance of nitrogen-cycle-related genes was based on the correspondence between KEGG (ver. 94.2, http://www.genome.jp/kegg/, 17 August 2024) and UniRef90 (mainly from LinkDB).

### 2.5. Data Analyses

Prior to analysis, logarithmic or square root conversion was performed on soil physicochemical properties, and the OTU table was flattened. R-4.1.2 was used to carry out the graphics and statistical analyses in this study. One-way analysis of variance (ANOVA) and multiple comparisons using Duncan’s method were conducted to analyze the physicochemical properties, microbial diversity, and functional difference of the rhizospheric soil using SPSS Statistics 23.0. In order to study the species composition and diversity information of the samples, all valid sequences of all samples with Kraken2 (parameter–confidence 0.2) were annotated and classified. Biomarkers of different groups were defined using LEfSe analysis to identify biological markers with significant differences between groups. A threshold logarithmic LDA score of 4.0 was applied. Non-metric multi-dimensional scaling (NMDS) and principal coordinate analysis (PCoA) based on the Bray–Curtis distance were employed to examine the differences in the gene composition among treatments. Metagenomic data were compared and annotated with Level3 pathway information in KEGG database to explore the relationship between species of microbiome and nitrogen metabolism. DiTing (version 0.9) software was used to infer and compare biogeochemical pathways in metagenomic data. Pearson’s correlation analysis was used to reveal the correlations between N-cycle-related processes, normalized abundances of N-cycle-related genes, and soil properties, and the correlations were visualized in heatmap plots.

## 3. Results

### 3.1. Responses of Rhizospheric Soil Nutrients to TCMR Application

TCMR significantly enhanced the total nutrient content of the rhizospheric soil. C1, MC, and WC significantly increased the contents of TP, TN, and NO_3_^−^-N, while NH_4_^+^-N contents decreased, compared with CK. When combined with fumigants, the contents of TP, TN, and NO_3_^−^-N were elevated. TCMR application (C1, MC, and WC) showed higher TP, TN, and NO_3_^−^-N contents compared to fumigants treatment (W1 and M1). TCMR application also improved total soil enzyme activity, with W1 showing lower activity than WC and M1 showing lower activity than MC (Table 1).

### 3.2. Responses of the Rhizosphere Microbial Community Composition and Diversity to TCMR Application

**Figure 1:**
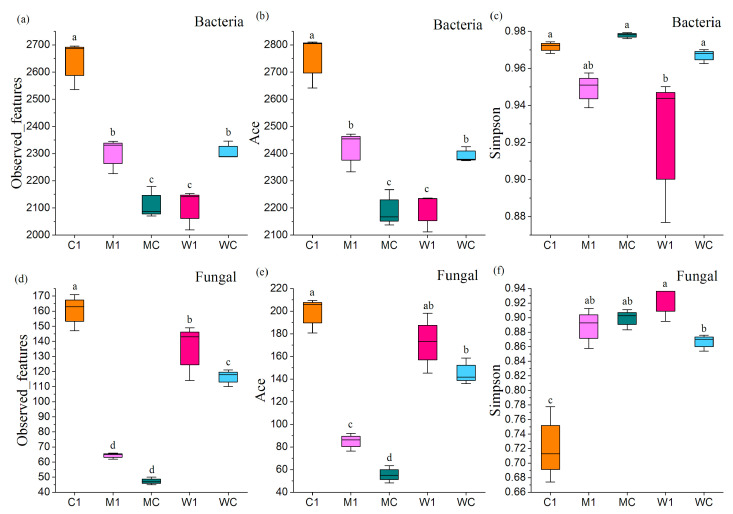
α diversity of rhizosphere bacterial (**a**,**c**,**e**) and fungal (**b**,**d**,**f**) microbial communities under TCMR application. C1: unfumigated soil with TCMR application; M1: dazomet fumigation without TCMR application; MC: TCMR was applied after dazomet fumigation; W1: metam-sodium fumigation without TCMR application; WC: TCMR was applied after metam-sodium fumigation. Lowercase letters denote significant differences based on the Duncan’s multiple comparisons of different treatments (*p* < 0.05).

**Figure 2:**
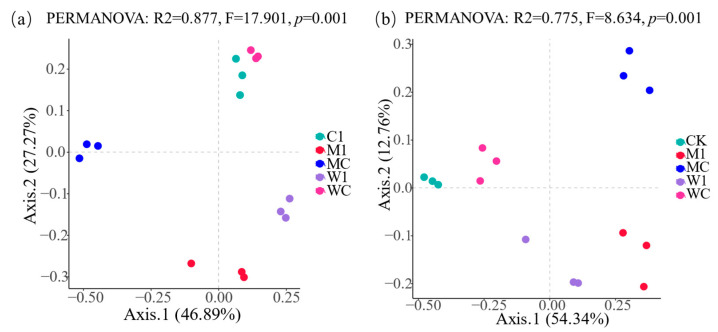
Principal coordinate analysis (PCoA) based on Bray–Curtis distances of rhizosphere bacterial (**a**) and fungal (**b**) microbial communities under different treatments. C1: unfumigated soil with TCMR application; M1: dazomet fumigation without TCMR application; MC: TCMR was applied after dazomet fumigation; W1: metam-sodium fumigation without TCMR application; WC: TCMR was applied after metam-sodium fumigation.

**Figure 3:**
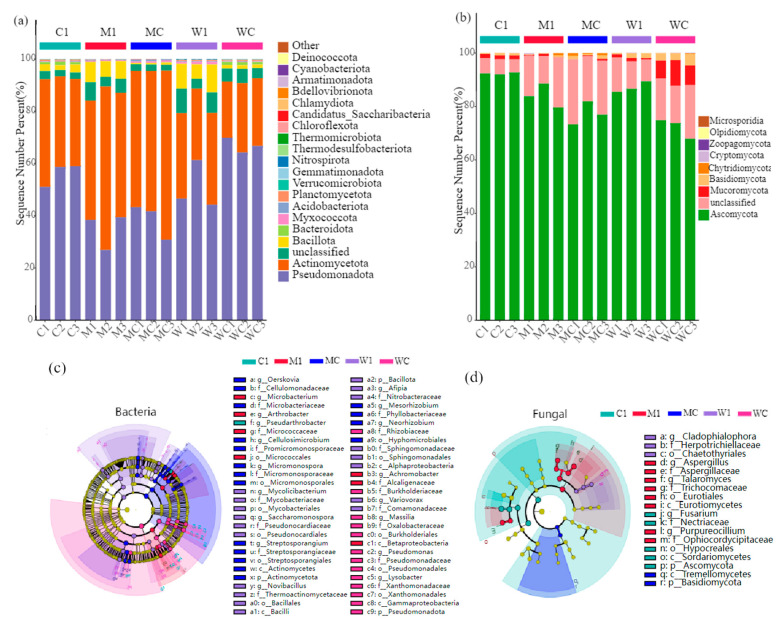
Keystone species composition of rhizosphere bacterial (**a**) and fungal (**b**) microbial communities at phylum level under different treatments. Lefse analysis further identified distinct species of rhizosphere bacteria (**c**) and fungi (**d**) from different treatments; LDA(log 10) ≥ 4.0, p, c, o, f, and g represent phylum, class, order, family and genus, respectively; C1: unfumigated soil with TCMR application; M1: dazomet fumigation without TCMR application; MC: TCMR was applied after dazomet fumigation; W1: metam-sodium fumigation without TCMR application; WC: TCMR was applied after metam-sodium fumigation.

TCMR application significantly impacted the α diversity of bacterial and fungal microbial communities, as illustrated in Figure 1a–f. However, not all effects were statistically significant. The results of PCoA indicated that TCMR application significantly influenced the composition of the rhizosphere microbial community (*p* < 0.01) (Figure 2a,b). At the phylum level, Pseudomonadota, Actinomycetota, and Bacillota were identified as dominant bacterial populations in the rhizosphere. Similarly, Ascomycota dominated the rhizosphere fungal community, with a notable increase in Pseudomonadota following TCMR application. Beneficial bacteria, such as Mesorhizobium, significantly increased in relative abundance, while pathogenic bacteria like Afipia decreased at the genus level (Figure 3a,b). LEfSe analysis revealed 23 genus-level biomarkers, including significant differences in the abundance of species such as Pseudarthrobacter, Achromobacter, Mesorhizobium, Afipia, and Pseudomonas (Figure 3c,d).

### 3.3. Relationships Among Rhizosphere Soil Nutrients, Keystone Species, and Community Building

**Figure 4:**
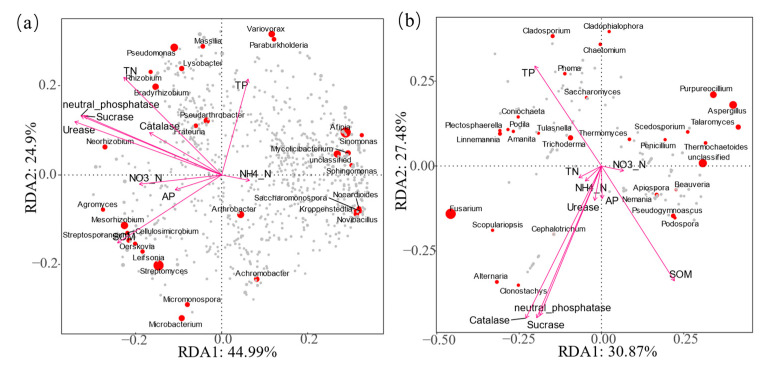
Redundancy analysis of rhizosphere soil nutrients and keystone species of rhizospheric bacteria (**a**) and fungi (**b**) (*p* < 0.001).

RDA analysis mainly relies on the R language VEGAN package and the visualization with ggplot2. The result showed that rhizospheric soil nutrients explained 69.89% and 58.35% of the keystone species variation in bacteria and fungal, respectively, and TN, TP, and urease significantly affected the composition of bacterial keystone species (Figure 4). TN and urease significantly influenced the composition of bacterial keystone species, while TP significantly influenced the composition of fungal keystone species. That is, soil nutrients regulated the microbial community construction process by affecting the community change of microbial keystone species. However, this result was not observed in the construction of fungal communities, which indicated that the process of construction of bacterial and fungal communities had different mechanisms. The abundance of keystone species was significantly correlated with TN, urease, TP, NO_3_^−^-N, and NH_4_^+^-N. TN, urease, and TP were identified as keystone factors affecting microbial community construction and composition (*p* < 0.001).

### 3.4. Analysis of Nitrogen Metabolic Activity in Various Different Conditions

**Figure 5:**
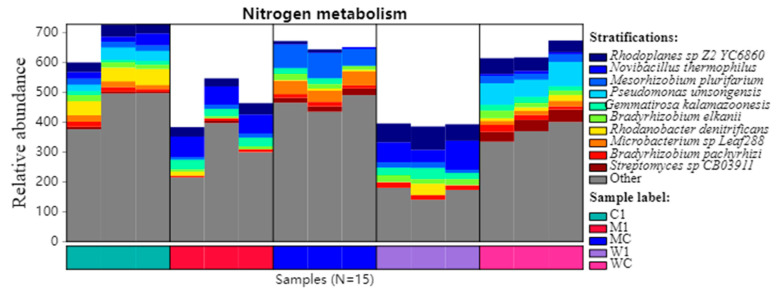
Analysis of nitrogen metabolism of rhizosphere microbial communities after different treatments based on KEGG pathway level 3 (*p* < 0.001). C1: unfumigated soil with TCMR application; M1: dazomet fumigation without TCMR application; MC: TCMR was applied after dazomet fumigation; W1: metam-sodium fumigation without TCMR application; WC: TCMR was applied after metam-sodium fumigation.

Further analysis of nitrogen metabolism was conducted at KEGG pathway level 3 (Figure 5). The top 10 microbial communities involved in nitrogen metabolism were identified, including Rhodoplanes, *Novibacillus thermophilus*, Mesorhizobium, Pseudomonas, Gemmatirosa kalamazoonesis, Microbacterium, *Bradyrhizobium pachyrhizi*, and *Streptomyces sp.* CB03911. TCMR application increased the abundance of *S. sp.* CB03911, *B. pachyrhizi*, and Pseudomonas might be a species source that promotes nitrogen metabolism, while Rhodoplanes was associated with a decrease.

### 3.5. Responses of Functional Genes and Factors of Nitrogen Cycle in TCMR Application

**Figure 6:**
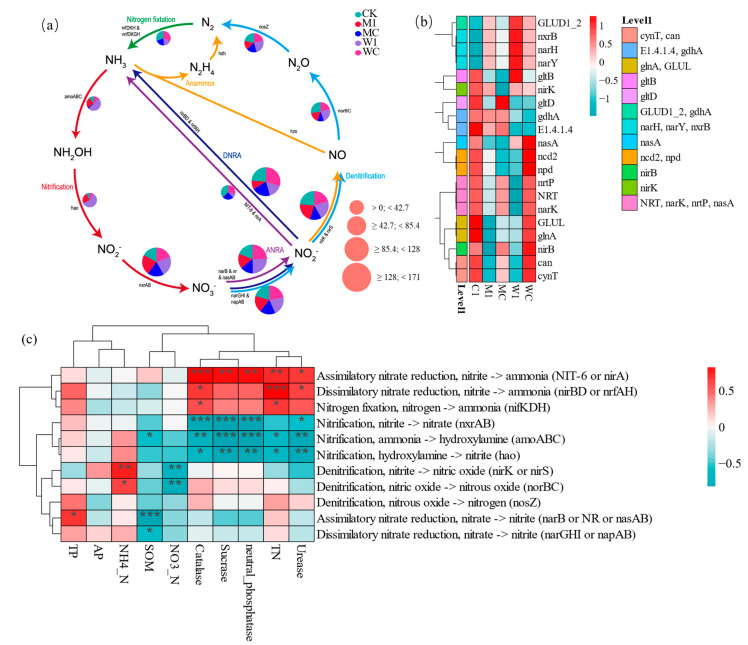
Schematic diagram of nitrogen cycle in rhizosphere soil (**a**). Abundance of functional genes and factors of nitrogen cycle (**b**). Relationship between functional genes and factors of nitrogen cycle in rhizosphere soil (**c**). C1: unfumigated soil with TCMR application; M1: dazomet fumigation without TCMR application; MC: TCMR was applied after dazomet fumigation; W1: metam-sodium fumigation without TCMR application; WC: TCMR was applied after metam-sodium fumigation. * *p* < 0.05; ** *p* < 0.01; *** *p* < 0.001.

Different treatments had distinct impacts on soil microorganisms, with a multitude of functional genes involved in the nitrogen cycle. The KO abundance table revealed six nitrogen metabolic pathways, primarily denitrification and dissimilatory nitrate reduction pathways. Abundant gene clusters related to nitrogen cycling included *narGHI*, *napAB*, *nirK*, *nirS*, *nirBD*, and *nrfAH* (Figure 6a). The total abundance of genes such as *can*, *cynT*, *nirB*, *glnA*, *GLUL*, *narK*, *NRT*, and *nrtP* significantly increased with TCMR treatments (MC, WC, C1), but not with M1 and W1, while the total abundance of *nxrB*, *nirK*, *narγ*, *narH*, and *GLUD1_2* significantly decreased (Figure 6b). Correlation analysis revealed that TN and urease were positively correlated with *nirB*, *nirA*, and *nifHDK* but negatively correlated with *amoABC* and *hao*. NO_3_^−^-N was negatively correlated with *nirK* and *norBC* genes, while NH_4_^+^-N was positively correlated with these genes. TP was positively correlated with *narB* and *nasA* (Figure 6c).

## 4. Discussion

### 4.1. Response of the Microbial Community Construction to Added TCMR and Its Influencing Factors

The establishment and maintenance of soil biomes is an intricate process involving the interplay of multiple factors [15,16]. Fumigants not only directly impact crop yield but also indirectly influence plant productivity through their effects on the soil microbiome [17]. The application of bio-organic fertilizer after chemical fumigation is critical for ensuring a balanced nutrient supply and promoting the recovery of soil microbial communities, which is essential for restoring rhizomatic immune barriers [18]. Our findings indicate that TCMR application significantly influences the construction of the rhizosphere microbial community. Different fumigants exert varying effects on soil microorganisms. Compared to dazomet fumigation, metam-sodium fumigation led to a significant reduction in bacterial diversity while causing a notable increase in the fungal community, consistent with previous research [19]. The addition of TCMR substantially promoted bacterial community construction but did not significantly affect the fungal community. Keystone species in the bacterial community primarily belonged to Pseudomonadota, Actinomycetota, and Bacillota [20,21,22]. Notably, the relative abundance of beneficial bacteria such as Mesorhizobium increased, while pathogenic bacteria such as Afipia decreased. Additionally, Actinomycetota played a role in decomposing organic matter, regulating the soil microenvironment, and maintaining soil ecological balance by reducing pathogenic bacteria [23]. Ascomycota was the dominant fungal population at the phylum level, and TCMR application increased their relative abundance. This could be linked to the presence of eutrophic fungi, which are associated with enhanced soil fertility [24].

We observed that bacterial community composition was predominantly influenced by soil nutrient levels, with TN, SOM, and urease playing significant roles in shaping bacterial communities, consistent with previous research [25]. The difference is that TP, SOM, and TN significantly affected the composition of keystone fungal species, which is not consistent with the conclusions of some studies [26,27,28]. These may be attributable to differences in land use and fertilization practices. Additionally, distinct mechanisms underlie the construction of bacterial and fungal communities [29]. In future investigations, factors such as root exudates, root morphology, and pot planting should be taken into consideration.

Moreover, we found that TCMR treatment led to a significant increase in TN, TP, NO_3_^−^-N, and urease levels in the rhizosphere soil, and a significant decrease in NH_4_^+^-N (Table 1, Figure 4). In soil, urease catalyzes the breakdown of urea to produce amines. Although amines is the preferred nitrogen source for both microbes and plants, nitrogen fixation, nitrite reductase, and the nitrate/nitrite transport systems are controlled by amines induced inhibition, which can ultimately affect crop yields [30]. TCMR might regulate nitrogen metabolism through affecting amines metabolism. These factors not only influenced the microbial functional model for carbon and phosphorus cycling but also played a pivotal role in shaping nitrogen cycling functional gene combinations [31]. Our results suggest that microbial functional traits related to soil nutrient cycling may be highly responsive to soil nitrogen bioavailability [32]. The increased abundance of nitrogen-metabolizing microbial communities, such as Rhodoplanes, *N. thermophilus*, Mesorhizobium, and others, further supports this conclusion.

### 4.2. Responses of Keystone Genes in the Nitrogen Cycle to Added TCMR and Their Correlations with the Microbial Community Structure

Microbial activity is essential in regulating nitrogen conversion, involving six classical processes: nitrogen fixation, nitrification, denitrification, assimilatory nitrate reduction (ANRA), dissimilatory nitrate reduction (DNRA), and ammonification [33,34]. In this study, we identified six nitrogen metabolic pathways, with denitrification and DNRA being the most prominent. Consistent with previous research [6,35], the combined application of TCMR and fumigation increased the abundance of denitrification genes relative to fumigation alone. Heterotrophic microorganisms, which are primarily responsible for denitrification, benefit from the addition of organic materials as these enhance nutrient availability, promoting the growth of denitrifying microorganisms [36,37]. Furthermore, the DNRA pathway was found to mitigate nitrogen loss by converting soil nitrate into ammonium nitrogen. The combined application of TCMR and fumigation not only regulated soil PH and reduced heavy metal content but also facilitated the rapid restoration of nitrogen cycle processes in the soil ecosystem [38,39].

The changes in microbial functional genes reflect the internal driving forces of soil nitrogen cycling. Under TCMR application, the reduction of NO_3_^−^ to NO_2_^−^ was significantly enhanced, primarily catalyzed by membrane-bound nitrate reductases (NAR, *narG*), periplasmic nitrate reductases (NAP, *napA*), or assimilation nitrate reductase (NAS, *nasA*, *nirA*) [38,39,40]. Additionally, TCMR fertilization significantly increased the abundance of carbonic anhydrase genes (*can*, *cynT*), which are involved in CO_2_ capture and the promotion of calcium carbonate precipitation through heterotrophic denitrification [41]. Interestingly, the application of TCMR led to a marked increase in the abundance of DNRA-related gene *nirB* while decreasing the abundance of the denitrification gene *nirK*. The genes *nirB* and *nirK* encode nitrite reductases, which are crucial for nitrogen metabolism. Gene *nirB* also plays a role in nitrogen assimilation [42]. Amines inhibition regulates the expression of the structural gene for nitrite reductase during nitrogen assimilation. The application of TCMR reduces amines concentrations in the rhizosphere, leading to an increase in the abundance and activity of gene *nirB*. This not only accelerates nitrogen conversion but also enhances nitrogen uptake and utilization. These findings diverge from some previous studies [43,44,45], potentially indicating unique interactions between nitrogen cycling genes and TCMR treatments.

The combined application of TCMR and fumigation significantly enriched soil TN, and such treatment was positively associated with genes like *nirB*, *nirA,* and *nxrAB*, and negatively correlated with *hao* and *amoABC*. These linkages suggest that TCMR application inhibits microbial nitrogen assimilation while promoting microbial nitrogen mobilization, resulting in an accumulation of bioavailable nitrogen. The addition of TCMR and the accumulation of bioavailable nitrogen after fumigation were significantly correlated with soil nitrogen content in the whole habitat. This may suggest that *nirB* and *nirK* are keystone genes regulating soil nitrogen cycling process, promoting the mobilization of nitrogen in continuous cropping, improving species diversity after fumigation, and promoting the sustainable use of nitrogen.

## 5. Conclusions

The combined application of TCMR after chemical fumigation substantially aids in the reconstruction of bacterial communities while having no obvious effect on fungal populations. This treatment fosters an increase in microbial species diversity and promotes the abundance of functional genes related to nitrogen cycling. Specifically, the diversity and abundance of denitrification and DNRA genes were positively influenced by TCMR application. Soil organic nitrogen was found to be closely associated with keystone genes such as *nirB* and *nirK*, which regulate microbial nitrogen uptake and transport. Moreover, TCMR fertilization led to the enrichment of the *can* and *cynT* genes, which are involved in microbial mineralization processes. These genes promote the precipitation of calcium carbonate through heterotrophic denitrification and CO_2_ capture, highlighting the coupling of microbial metabolic processes. In conclusion, the application of TCMR following fumigation effectively promotes the construction of the soil microbial community and significantly influences the functional dynamics of the soil nitrogen cycle, ultimately enhancing the accumulation of bioavailable nitrogen, and increasing crop yield.

## Figures and Tables

**Table 1:** Effects of different treatments on rhizospheric soil nutrients.

Treatment	pH	SWC/(%)	SOM/ (g·kg^−1^)	TN/ (g·kg^−1^)	TP/ (g·kg^−1^)	AP/ (g·kg^−1^)
CK	6.54a	18.62a	42.98 ± 1.51a	0.96 ± 0.03a	0.18 ± 0.01a	2.40 ± 0.01a
C1	7.13a	19.86a	41.53 ± 0.88a	1.12 ± 0.06b	0.37 ± 0.18b	4.54 ± 0.18b
M1	7.1a	25.66a	37.91 ± 2.50b	1.03 ± 0.11a	0.37 ± 0.18b	5.20 ± 0.28c
MC	7.35a	23.04a	46.42 ± 0.92c	1.17 ± 0.08b	0.48 ± 0.10b	3.88 ± 0.45b
W1	7.1a	20.56a	35.94 ± 0.13d	1.10 ± 0.02a	0.70 ± 0.16c	2.74 ± 0.56a
WC	7.4a	21.46a	35.48 ± 0.92d	1.18 ± 0.01b	0.86 ± 0.10d	4.97 ± 0.29b
**Treatment**	**NO_3_^−^-N/(mg·kg^−1^)**	**NH_4_^+^-N/(mg·kg^−1^)**	**Urease/** **(μg·g^−1^·d^−1^)**	**Catalase/(U·g^−1^)**	**Sucrase/** **(mg·g^−1^·d^−1^)**	**Neutral phosphatase/(μmoL·g^−1^·d^−1^)**
CK	15.31 ± 0.16a	2.34 ± 0.15a	1354.32 ± 5.11a	36.23 ± 0.12a	29.52 ± 0.08a	1.96 ± 0.02a
C1	19.42 ± 0.05b	1.98 ± 0.25b	1537.62 ± 5.78b	37.04 ± 0.11a	33.72 ± 0.10b	2.17 ± 0.03b
M1	20.32 ± 0.18b	1.93 ± 0.08b	1146.99 ± 2.47c	26.32 ± 0.09b	23.16 ± 0.12c	1.55 ± 0.01c
MC	25.31 ± 0.14c	1.67 ± 0.17c	1898.11 ± 3.08d	30.63 ± 0.13c	29.14 ± 0.17a	1.93 ± 0.02a
W1	19.90 ± 0.16b	1.84 ± 0.10b	1214.76 ± 2.49d	26.69 ± 0.04b	21.34 ± 0.06c	1.46 ± 0.01d
WC	23.58 ± 0.21d	1.73 ± 0.11c	1781.55 ± 9.00d	28.75 ± 0.07d	28.46 ± 0.08a	1.87 ± 0.03a

Note: CK: rhizosphere soil without any treatment; C1: unfumigated soil with TCMR application; M1: dazomet fumigation without TCMR application; MC: TCMR was applied after dazomet fumigation; W1: metam-sodium fumigation without TCMR application; WC: TCMR was applied after metam-sodium fumigation. Lowercase letters denote significant differences based on the Duncan’s multiple comparisons of different treatments (*p* < 0.05).

## Data Availability

Data from the sequencing were deposited in the National Microbiology Date Center (https://www.cncb.ac.cn/, 19 November 2024) (No. SUBCRA033236).
